# Genome-wide association analysis of milk production, somatic cell score, and body conformation traits in Holstein cows

**DOI:** 10.3389/fvets.2022.932034

**Published:** 2022-10-04

**Authors:** Peng Wang, Xue Li, Yihao Zhu, Jiani Wei, Chaoxin Zhang, Qingfang Kong, Xu Nie, Qi Zhang, Zhipeng Wang

**Affiliations:** ^1^Heilongjiang Animal Husbandry Service, Harbin, China; ^2^College of Animal Science and Technology, Northeast Agricultural University, Harbin, China; ^3^Bioinformatics Center, Northeast Agricultural University, Harbin, China; ^4^School of mathematics, University of Edinburgh, Edinburgh, United Kingdom; ^5^College of Animal Science and Technology, China Agricultural University, Beijing, China

**Keywords:** milk production traits, body conformation traits, pleiotropic effect, genome-wide association study, Holstein cattle

## Abstract

Milk production and body conformation traits are critical economic traits for dairy cows. To understand the basic genetic structure for those traits, a genome wide association study was performed on milk yield, milk fat yield, milk fat percentage, milk protein yield, milk protein percentage, somatic cell score, body form composite index, daily capacity composite index, feed, and leg conformation traits, based on the Illumina Bovine HD100k BeadChip. A total of 57, 12 and 26 SNPs were found to be related to the milk production, somatic cell score and body conformation traits in the Holstein cattle. Genes with pleiotropic effect were also found in this study. Seven significant SNPs were associated with multi-traits and were located on the *PLEC, PLEKHA5, TONSL, PTGER4*, and *LCORL* genes. In addition, some important candidate genes, like *GPAT3, CEBPB, AGO2, SLC37A1*, and *FNDC3B*, were found to participate in fat metabolism or mammary gland development. These results can be used as candidate genes for milk production, somatic cell score, and body conformation traits of Holstein cows, and are helpful for further gene function analysis to improve milk production and quality.

## Introduction

Milk is a source of nutrients essential for human growth and development. The milk production traits are important for the dairy industry. Body conformation traits have been applied in several countries with the development of dairy cattle breeding since they are closely related to the health (1), productivity (2), lifetime (3), and calving ease (4) of cows. Some studies have identified the genetic correlation between body conformation traits and first lactation milk yield to be between 0.48 and 0.54 (5). These correlations are therefore very important for the dairy industry to improve the milk production traits and body conformation traits.

The rapid development of sequencing technology has revealed the cause variants of complex traits using genome-wide association analysis (GWAS). A study by Schennink et al. (6) has revealed *DGAT1* and *SCD1* to be highly associated with the composition of milk-fat (long-chain fatty acid). Kiser et al. (7) verified the TFAP2A gene to be related to the production of colostrum in Jersey cattle. It reported the genes *CDH2* and *GABRG2* to be related to the milk fat percentage and milk protein traits, respectively, in dual-purpose Xinjiang brown cattle (8). Bouwman et al. (9) and Vanvanhossou et al. (10) have reported the VEPH1 gene to be associated with conformation. However, the identified genes have not explained all genetic variances. There is a need to continue the search for novel genes related to some quantitative traits.

This study conducted GWAS using the Illumina Bovine HD100k(100k) BeadChip, for identifying important candidate genes or variants related to milk production, somatic cell score, and body conformation traits. There was an expectation for discovering novel genetic variations or candidate genes.

## Materials and methods

### Animal population

This experiment involved 1,313 cows from 7 different pastures in Heilongjiang Province. The use and care of the animals in this study were approved by the Animal Care Advisory Committee, Northeast Agricultural University (Harbin, China), and all the experimental procedures were according to the university's guidelines for animal research.

### Genotypes data

The samples were collected from the tail roots near the hips of the cows. The DNA in the hair was extracted and genotyped using Illumina Bovine HD100k BeadChip, containing 95,256 SNPs. The markers with minor allele frequencies <0.05 and call rates <0.90 were filtered out and individuals with a call rate of 0.80 or greater were selected. These SNPs were distributed across 29 chromosomes.

### Population stratification

**Figure 1:**
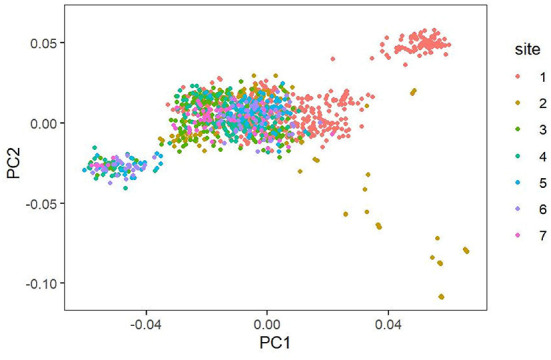
Population structure demonstrated by principal component analysis.

The SNP genotypes of these individuals were used to estimate the population stratification based on principal component analysis (PCA), and Plink (version 1.9) (11) was used to analyze a total of 1,310 cows with 86,645 markers covering the whole genome to study the population structure (12). The software uses the default matrix construction method to construct G matrix and get the PCA results. We used R language (version 4.1.2)—ggplot 2 package to draw pictures. The PCA scatterplots (Figure 1) illustrate a clear population structure for the 1,310 individuals in the seven pastures cattle herds that comprised our study population.

**Figure 2:**
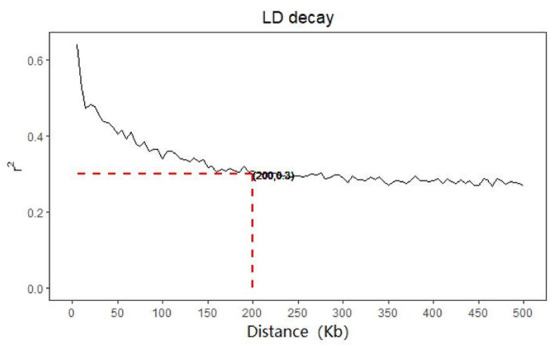
LD decay of cow.

### Genome-wide association analysis

Combination with dairy herd improvement data of National Holstein cows in China, this study estimated the genomic estimated breeding values (GEBVs) of all animal milk production traits, somatic cell score, and body conformation traits, using single-step genomic best linear unbiased prediction (ssGBLUP). The ssGBLUP was developed to integrate all the information including genotypes, phenotypes, and pedigree information in one step, and each SNP effect was calculated using the FarmCPU method (13) based on the predicted GEBVs. The ssGBLUP method is an improvement of BLUP, in which the pedigree relationship matrix a^−1^ matrix must be replaced by H^−1^ (14). The specific model is as follows:


$$\begin{array}{l} \text{y}=X\text{b}+Z\text{u}+\text{e} \end{array}$$

$$\begin{array}{l} H=\left[\begin{array}{cc} H_{11} & H_{12}\\ H_{21} & H_{22} \end{array}\right]\\ =\left[\begin{array}{cc} A_{11}+A_{12}A_{22}^{-1}(G-A_{22})A_{22}^{-1}A_{21} & A_{12}A_{22}^{-1}G\\ GA_{22}^{-1}A_{21} & G \end{array}\right] \end{array}$$

Where y was each phenotypic value vector; b is the fixed effect of the field and the PCA effect to explain the population stratification, and u is a vector of animal effects. The e was a vector of random residual effects with e~N(0,I), and X, Z were incidence matrices for b and u, respectively.


$$\begin{array}{l} H^{-1}=A^{-1}\\ \;+\left[\begin{array}{cc} 0 & 0\\ 0 & \tau {\left[(1-\text{w})(\alpha +\text{b}*\text{G})+\text{w}*{\text{A}}_{22}\right]}^{-1}-\omega {\text{A}}_{22}^{-1} \end{array}\right] \end{array}$$

Where, the A matrix is pedigree relationship matrix, A_22_ is a numerator relationship matrix for genotyped animals, and G is a genomic relationship matrix (15). G^−1^ was obtained as the inverse of a combination of the G matrix and the corresponding A matrix. The w is the weight of *A*_22_ in the matrix, the default value is 0.05. The τ and ω are 1. We use DMU software to calculate the GEBV value. Both G and H matrices were derived using software default parameter setting by DMU software. G was calulated as:


$$\begin{array}{l} G={\frac{WDW^{{}^{\prime }}}{2\sum_{i=1}^{n}p_{i}\left(1-p_{i}\right)}}_{} \end{array}$$

Where p_*i*_ is the allele frequency at locus i in all genotyped animals, is a normalizing constant (16) that sums expected variances across markers scaling G toward the A matrix (17), D is weight for each locus(I if same variance assumed), W is a design matrix as follows):


$$w_{ii}\{\begin{array}{c} 0-2p_{i},homozygous\\ 1-2p_{i},heterozygous\\ 2-2p_{i},homozygous \end{array}$$

Each SNP effect was calculated using the FarmCPU method (13) based on the predicted GEBVs. The FarmCPU method (13) in this study can be written as two models.


$$\begin{array}{l} \begin{array}{c} y=SNP_{\text{i}}+K+e\\ y=pseudQTN+SNP_{\text{i}}+e \end{array} \end{array}$$

The y is the GEBV value. The pseudoQTN is significant marker from previous loops that is null when the model begins. SNP_i_ is testing marker in each loop. The K is the kinship between each individuals. The e is residual vector.

For each trait, the threshold *P*-value for genome-wide significance was 5.99 × 10^−7^= 0.05/83446 using the Bonferroni multiple test method.

### QTLs annotation analysis

The cattle QTL data were downloaded from the Cattle QTL database (https://www.animalgenome.org/cgi-bin/QTLdb/BT/index) referred to as the ARS-UCD1.2 assembly. The square of the correlation coefficient (r^2^) between the two loci is used to evaluate the range of LD measurement, because r^2^ is considered to be more robust and not affected by changes in allele frequency and population size (18). Haploview software was used to calculate the genotype correlation coefficient (r^2^) between all SNP pairs in the cow population to estimate the LD of the whole genome, and the LD decay map with distance of the cow population was visualized.

## Results

### Population stratification

The phenomenon of group stratification is an important research problem in the study of group association (19). In order to determine the population stratification level, we drew the population structure by principal component analysis (PCA). The PCA scatterplots shows the population structure of a 1,300 individual composed of seven pastures (Figure 1). Different colors represent different pastures. It can be seen that it is mainly divided into three clusters, but most of the cows in the seven pastures are gathered together, and only a few cows are separated. These clusters indicate that, although individuals may come from different ranches, they still retain close genetic relationships.

### The genome-wide association study

Basic descriptive statistics of milk production traits, somatic cell score and body conformation traits (see Table 1). A total of 86,645 SNPs were retained after quality control for the GWAS (Table 2). The average physical distance between the adjacent SNP markers was approximately 29.58 kb, ranging between 26.37 kb (BTA19) and 32.02 kb (BTA8).

**Table 1:** Descriptive statistics of milk procuction trants and body conformation traits.

**Statistic**	**MY (kg)**	**FP (%)**	**PP (%)**	**SCS**	**BFCI**	**FTLEG**	**DCCI**
Mean	8382.99	3.85	3.31	4.02	85.68	85.46	85.91
Standard Deviation	1950.68	0.50	0.26	1.44	4.88	4.16	7.68
Minimum	1505.00	2	2.17	1.00	65.25	65.80	56.18
Maximum	15983.00	6.20	5.00	9.00	98.36	99.00	99.95
Coefficient of Variation	0.23	0.13	0.08	0.36	0.06	0.05	0.09

**Table 2:** Distribution of SNPs after quality control.

**BTA**	**Length (Mb)**	**No. SNP** **(Chip data)**	**No. SNP** **(after QC)**	**Density** **length/SNP(kb)**
1	158.53	5556	5188	30.56
2	136.23	4688	4367	31.20
3	121.01	4508	4158	29.10
4	120.00	4049	3760	31.92
5	120.09	4523	4083	29.41
6	117.81	4364	3977	29.62
7	110.68	3903	3551	31.17
8	113.32	3805	3539	32.02
9	105.45	3695	3469	30.40
10	103.31	3626	3376	30.60
11	106.98	3801	3522	30.38
12	87.22	3044	2842	30.69
13	83.47	3064	2822	29.58
14	82.40	3045	2796	29.47
15	85.01	3119	2885	29.47
16	81.01	2826	2586	31.33
17	73.17	2668	2506	29.20
18	65.82	2605	2389	27.55
19	63.45	2726	2406	26.37
20	71.97	2737	2498	28.81
21	69.86	2573	2374	29.43
22	60.77	2201	2038	29.82
23	52.50	2110	1951	26.91
24	62.32	2259	2081	29.95
25	42.35	1726	1589	26.65
26	51.99	1823	1708	30.44
27	45.61	1699	1624	28.09
28	45.94	1735	1630	28.18
29	51.10	1871	1731	29.52
Total	2489.37	90349	83446	29.58

**Figure 3:**
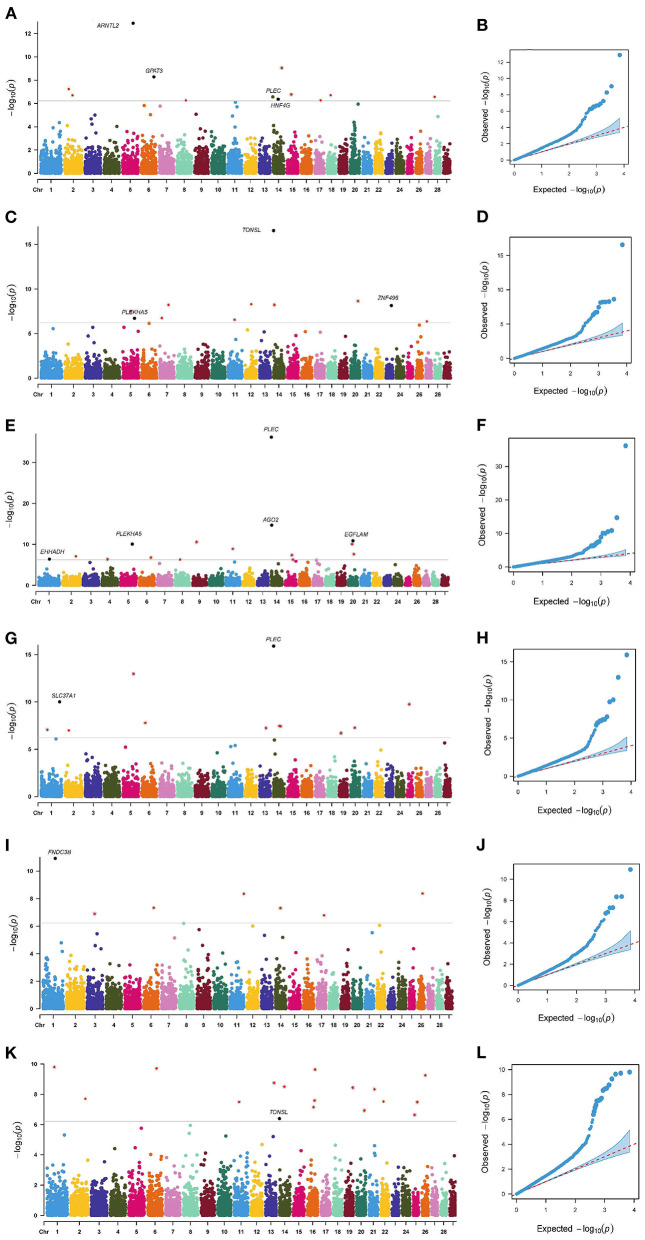
Manhattan plots and Quantile-Quantile plots for the milk production and somatic cell score traits. MY **(A,B)**, FY **(C,D)**, FP **(E,F)**, PP **(G,H)**, PY **(I,J)** and SCS **(K,L)**.

**Figure 4:**
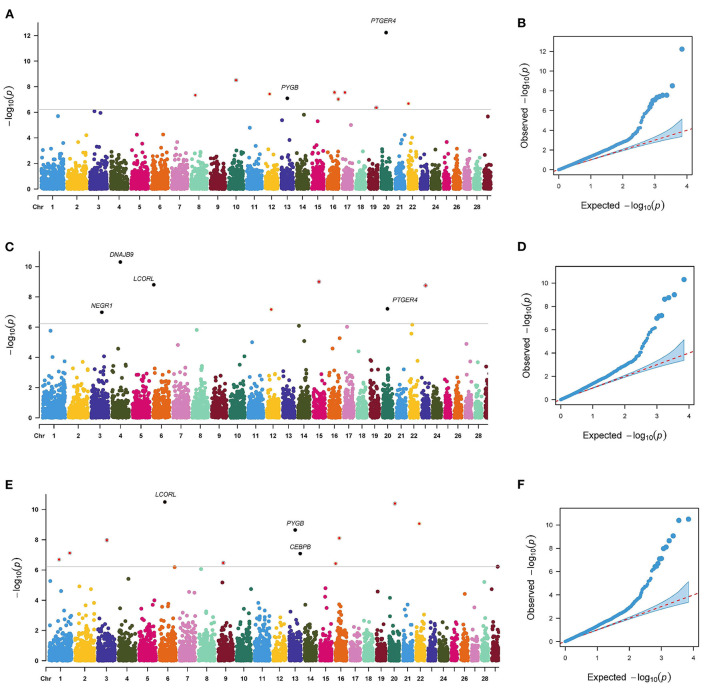
Manhattan plots and Quantile-Quantile plots for the body conformation traits. BFCI **(A,B)**, DCCI **(C,D)** and FTLEG **(E,F)**.

The *p*-value profiles of all the SNP markers associated with each trait are represented in Figures 3, 4 and included the Manhattan and Quantile-Quantile plots. In total, 95 genome-wide significant SNPs were detected for the milk production traits, such as milk yield (MY), milk fat yield (FY), milk fat percentage (FP), milk protein yield (PY), milk protein percentage (PP), somatic cell score (SCS), and body conformation traits (body form composite index, BFCI; daily capacity composite index, DCCI; feed and leg conformation, FTLEG). There were 57, 12 and 26 SNPs related to milk production, somatic cell score and body conformation traits, respectively. Among them, we mainly focused on the first few significant SNPs in each trait. In addition, we also found seven SNPs that overlap with multiple traits, such as *PLEC* is related to MY, FP and PP, *PLEKHA5* is related to FP and FY, *TONSL* is connected with FY and SCS, *LCORL* is correlated with DCCI and FTLEG, *PYGB* is related to BFCI and FTLEG, and *PTGER4* is related to BFCI, and DCCI (see Table 3).

**Table 3:** The SNPs and candidate genes with pleiotropic effect in this study.

**SNP name**	**Traits**	**Gene**	Distance(kb)*	**Gene full name**	**Gene function**
BovineHD1400000287	MY, FP, PP	PLEC	Intron	Plectin	Related to the MY, FP, and PP traits in Chinese Holsteins (20).
BovineHD0500025853	FP, FY	PLEKHA5	Intron	Pleckstrin homology domain containing, family A member 5	Significantly associated with FP (21).
BovineHD1400011649	MY, PY	HNF4G	Intron	Hepatocyte nuclear factor 4 gamma	Associated with childhood obesity (22). Key regulators of beef cattle carcass IMF (23).
BovineHD1400000206	FY, SCS	TONSL	1.65 (U)	Tonsoku like, DNA repair protein	Related to milk yield (24, 25) and affect the gamma–linolenic acid, long–chain saturated fatty acids and milk fat percent of the Canadian Holstein cows (26).
BovineHD4100004660	DCCI, FTLEG	LCORL	665.01 (D)	Ligand dependent nuclear receptor corepressor like	Affect human height (27), pig body length (28), horse height (29), chicken carcass weight (30), and the growth and development of cattle (31). Associated with the human skeletal frame size (32).
BovineHD1300012605	BFCI, FTLEG	PYGB	0.61 (D)	Glycogen phosphorylase B	Inhibition of glycogen utilization (33)
BTA−50244–no–rs	BFCI, DCCI	PTGER4	541.63 (D)	Prostaglandin E receptor 4	Relaxation to the smooth muscle (34), leading to the phosphorylation of glycogen synthase kinase−3 (35), involving in osteoporosis (36), and regulating lipid droplet size and mitochondrial activity in the white adipose tissue (37, 38).

*U, Upstream; D, Downstream.

As shown in Tables 4, 5, 12, 11, 15, 11, 12, and 17 genome-wide significant SNPs were detected for MY, FY, FP, PY, PP, and SCS, respectively. These significant SNPs are mainly distributed in BTA 1, BTA 2, BTA 5, BTA 6, BTA 11, BTA 14, and BTA 20, with as many as 9 SNPs on BTA 14.

**Table 4:** Genome-wide significant SNPs are associated with milk production traits.

**Traits**	**SNP name**	**BTA**	**Postion (Mb)**	**MAF**	**Nearest gene**	Distance (kb)*	***P*–value**	**SNP effect**
MY	BTB−00088434	2	33.86	0.0599	KCNH7	39.78 (U)	5.87E−08	−295.1002
MY	Hapmap40999–BTA−47831	2	62.78	0.4824	TMEM163	0.81 (D)	2.01E−07	−131.0532
MY	5–82810184–C–T–rs110495697	5	82.40	0.4156	ARNTL2	Intron	1.30E−13	206.5174
MY	BovineHD0600027996	6	98.51	0.2000	GPAT3	144.85(D)	5.23E−09	−188.5885
MY	BovineHD0800021118	8	69.70	0.2790	SLC39A14	Intron	5.53E−07	−145.3961
MY	BovineHD1400000287	14	0.88	0.2240	PLEC	Intron	2.71E−07	163.9574
MY	BovineHD1400011649	14	38.57	0.1725	HNF4G	Intron	4.36E−07	−154.3554
MY	BTA−07375–no–rs	14	66.16	0.3824	ERICH5	Intron	8.97E−10	194.9580
MY	BovineHD1500014407	15	49.22	0.3053	OR51L4	0.17(U)	1.23E−07	−155.6556
MY	BovineHD1700012968	17	45.45	0.2130	SFSWAP	90.47(D)	5.35E−07	−153.0319
MY	Hapmap55097–rs29010952	18	26.88	0.4260	GOT2	436.24(D)	1.95E−07	136.2285
MY	BovineHD2800000275	28	1.65	0.2076	URB2	45.14(D)	2.75E−07	−164.7354
FY	Hapmap24838–BTA−143176	5	62.15	0.4137	TMPO	560.66(U)	3.37E−08	3.4575
FY	BovineHD0500025853	5	90.66	0.3145	PLEKHA5	Intron	1.91E−07	3.4453
FY	ARS–BFGL–NGS−10921	7	14.97	0.3893	PDE4A	Intron	1.80E−07	3.3773
FY	BovineHD0700020203	7	67.04	0.3893	SGCD	536.03(U)	6.02E−09	−3.6651
FY	ARS–BFGL–NGS−75350	11	49.90	0.1893	TCF7L1	19.24(D)	2.79E−07	−3.8169
FY	ARS–BFGL–NGS−29737	12	47.06	0.1004	CXCL14	11.84(U)	5.06E−09	6.6175
FY	BovineHD1400000206	14	0.49	0.2172	TONSL	1.65(U)	2.76E−17	6.3376
FY	ARS–BFGL–NGS−55227	14	5.69	0.4179	KHDRBS3	711.31(U)	6.01E−09	3.7919
FY	ARS–BFGL–NGS−115233	20	58.83	0.4576	TRIO	Intron	2.29E−09	3.6663
FY	BovineHD2300011633	23	40.58	0.1126	ZNF496	Intron	7.11E−09	−5.8266
FY	ARS–BFGL–NGS−95089	27	8.94	0.2202	AGA	238.44(D)	4.30E−07	−3.7871
FP	BTB−00035766	1	81.92	0.3565	EHHADH	Intron	3.93E−07	0.0443
FP	Hapmap40546–BTA−48622	2	104.36	0.4168	MARCHF4	Intron	7.92E−08	−0.0460
FP	BovineHD0400011775	4	42.79	0.4855	OR2AV14	10.61(U)	4.24E−07	−0.0432
FP	BovineHD0500025853	5	90.66	0.3145	PLEKHA5	Intron	8.76E−11	0.0609
FP	Hapmap35196–BES10_Contig207_566	6	90.84	0.0679	SDAD1	Intron	1.73E−07	0.0917
FP	BovineHD0800011917	8	39.97	0.0832	SLC1A1	Intron	5.36E−07	0.0705
FP	ARS–BFGL–NGS−15823	9	28.53	0.3385	PKIB	Intron	2.70E−11	0.0611
FP	BovineHD1100015676	11	53.86	0.4229	–	–	1.28E−09	−0.0479
FP	BovineHD1400000287	14	0.88	0.2240	PLEC	Intron	6.48E−37	0.1423
FP	UA–IFASA−7269	14	3.10	0.2313	AGO2	Intron	2.01E−15	−0.1058
FP	BovineHD1500015438	15	52.73	0.1485	P2RY2	0.64(D)	4.08E−08	−0.0687
FP	BovineHD1500017563	15	60.50	0.4202	KCNA4	230.62(U)	5.11E−07	0.04461
FP	ARS–BFGL–BAC−27930	20	29.36	0.2080	DDX6	Intron	8.92E−11	−0.0795
FP	BTA−50420–no–rs	20	36.05	0.2107	EGFLAM	Intron	1.32E−11	−0.0872
FP	BTB−01263010	20	42.72	0.1565	CDH6	363.33(D)	2.61E−08	0.0769
PP	BovineHD0100013692	1	48.12	0.2420	–	–	8.70E−08	0.0222
PP	BovineHD0100041607	1	142.82	0.3676	SLC37A1	Intron	9.80E−11	0.0252
PP	ARS–BFGL–NGS−80635	2	31.59	0.4523	COBLL1	6.23(D)	1.02E−07	0.0182
PP	ARS–BFGL–NGS−117881	5	82.23	0.3859	C5H12orf71	21.60(U)	1.11E−13	−0.0318
PP	BovineHD0600008707	6	29.63	0.3897	BMPR1B	36.41(D)	1.62E−08	−0.0220
PP	ARS–BFGL–BAC−15734	13	48.97	0.3725	BMP2	179.62(U)	5.84E−08	−0.0210
PP	BovineHD1400000287	14	0.88	0.2240	PLEC	Intron	1.27E−16	0.0362
PP	BovineHD1400013724	14	46.18	0.4492	EXT1	Intron	3.55E−08	0.0211
PP	chr14_57250692	14	55.09	0.1481	NUDCD1	Intron	3.79E−08	−0.0283
PP	ARS–BFGL–NGS−21921	19	14.40	0.2817	CCL14	59.58(U)	1.97E−07	−0.0197
PP	BovineHD2000009361	20	32.69	0.2844	OXCT1	Intron	5.35E−08	−0.0294
PP	BovineHD2500004479	25	15.71	0.1275	XYLT1	Intron	1.78E−10	0.0365
PY	BovineHD0100027261	1	95.18	0.3279	FNDC3B	Intron	1.20E−11	3.3681
PY	BovineHD0300017107	3	56.64	0.3195	LMO4	54.14(U)	1.26E−07	−2.5817
PY	Hapmap26317–BTC−059618	6	80.53	0.1557	EPHA5	306.96(U)	4.69E−08	−3.2564
PY	ARS–BFGL–NGS−4974	11	106.56	0.1069	ZMYND19	5.22(D)	4.52E−09	−4.2183
PY	BovineHD1400011649	14	38.57	0.1725	CRISPLD1	226.89(D)	4.93E−08	3.2889
PY	BovineHD1700016449	17	55.89	0.4740	CCDC60	Intron	1.62E−07	−2.2566
PY	ARS–USMARC–Parent–EF034086–no–rs	26	37.90	0.4607	EMX2	63.30(D)	4.29E−09	2.6483

^*^U, Upstream; D, Downstream.

**Table 5:** Genome–wide significant SNPs are associated with somatic cell score.

**Traits**	**SNP name**	**BTA**	**Postion (Mb)**	**MAF**	**Nearest gene**	Distance(kb) *	**P value**	**SNP effect**
SCS	Hapmap59481–rs29019616	1	56.94	0.1893	GCSAM	Intron	1.59E−10	−0.1547
SCS	BovineHD0200033155	2	113.94	0.2847	NYAP2	90.45(D)	1.98E−08	0.1198
SCS	BovineHD0600020300	6	71.35	0.3580	CEP135	6.10(U)	1.94E−10	−0.1304
SCS	BovineHD1100011547	11	39.19	0.2080	CCDC85A	118.86(D)	3.15E−08	0.1376
SCS	BovineHD1300019252	13	67.15	0.2282	KIAA1755	21.69(D)	1.73E−09	−0.1423
SCS	BovineHD1400000206	14	0.49	0.2172	TONSL	1.65(U)	4.03E−07	−0.1159
SCS	BovineHD1400011508	14	38.01	0.2939	PI15	147.13(U)	3.10E−09	−0.1307
SCS	BovineHD1600013229	16	47.05	0.3996	ACOT7	Intron	7.12E−08	0.1065
SCS	BovineHD1600015783	16	55.27	0.0657	SERPINC1	Intron	2.54E−08	−0.2208
SCS	BTA−65815–no–rs	16	59.73	0.2267	RASAL2	Intron	2.31E−10	−0.1528
SCS	UA–IFASA−5305	19	59.21	0.1271	SOX9	289.75(D)	3.61E−09	−0.1751
SCS	BovineHD2000017315	20	61.61	0.4405	CTNND2	11.32(U)	1.18E−07	0.1036
SCS	BTA−52343–no–rs	21	42.73	0.1042	AKAP6	Intron	4.65E−09	−0.1536
SCS	Hapmap46118–BTA−108252	22	19.45	0.4435	GRM7	Intron	3.00E−08	−0.1121
SCS	ARS–BFGL–NGS−24519	25	10.59	0.1378	GSPT1	0.93(D)	2.31E−07	0.1394
SCS	ARS–BFGL–NGS−37189	25	32.40	0.07786	RCC1L	267.69(U)	3.28E−08	0.1948
SCS	Hapmap42542–BTA−40776	26	27.93	0.2504	SORCS1	20.95(D)	5.60E−10	0.1395

^*^U, Upstream; D, Downstream.

In addition, this study reported an interesting phenomenon where four SNPs were found to be related to multi-traits, including BovineHD0500025853 (BTA 5:90.66 Mb), BovineHD1400000206 (BTA 14:0.49 Mb), BovineHD1400000287 (BTA 14:0.88 Mb), and BovineHD1400011649 (BTA 14:38.57 Mb) (see Table 6). The bovinehd1400000287 SNP located in the 58th intron of the *PLEC* gene was found to be associated with MY, FP, and PP. The fat yield and the somatic cell score trait shared one SNP bovinehd1400000206 located 1.46 kb away from *TONSL* on BTA 14.

**Table 6:** Genome–wide significant SNPs are associated with body conformation traits.

**Traits**	**SNP name**	**BTA**	**Position (Mb)**	**MAF**	**Nearest gene**	Distance (kb)*	***P*-value**	**SNP effect**
BFCI	ARS–BFGL–NGS−39319	8	31.33	0.3836	MPDZ	122.75(D)	4.59E−08	−1.3217
BFCI	BovineHD1000015574	10	52.01	0.3450	AQP9	75.39(U)	3.07E−09	1.5722
BFCI	BovineHD1200008803	12	29.84	0.1481	HSPH1	19.83(U)	3.72E−08	1.9433
BFCI	BovineHD1300012605	13	42.81	0.4622	PYGB	0.61(D)	8.06E−08	1.2185
BFCI	ARS–BFGL–NGS−66252	16	50.24	0.0805	MMEL1	31.03(U)	2.79E−08	2.4780
BFCI	BovineHD1600023101	16	77.36	0.4538	ATP6V1G3	47.95(U)	9.44E−08	1.1905
BFCI	BovineHD1700005623	17	19.11	0.4050	SLC7A11	307.12(U)	2.82E−08	1.4169
BFCI	BovineHD1900015024	19	53.08	0.3546	RBFOX3	Intron	4.39E−07	1.2520
BFCI	BTA−50244–no–rs	20	34.30	0.3710	PTGER4	541.63(D)	5.84E−13	−1.9115
BFCI	BovineHD2200000513	22	1.99	0.1302	EOMES	123.32(U)	2.09E−07	1.8111
DCCI	BovineHD0300021562	3	73.79	0.4351	NEGR1	Intron	1.03E−07	−1.2304
DCCI	BTB−00190417	4	59.09	0.3496	DNAJB9	493.74(U)	4.93E−11	1.6662
DCCI	BovineHD4100004660	6	38.22	0.4271	LCORL	665.01(D)	2.39E−09	−1.4509
DCCI	ARS–BFGL–BAC−15023	12	31.34	0.4103	MTUS2	Intron	6.74E−08	1.2696
DCCI	BTB−00597065	15	41.00	0.3527	GALNT18	64.26(U)	9.91E−10	1.5227
DCCI	BTA−50244–no–rs	20	34.30	0.3710	PTGER4	541.63(D)	6.11E−08	−1.2173
DCCI	ARS–BFGL–NGS−97747	23	28.02	0.3840	CDSN	4.46(U)	1.77E−09	1.4608
FTLEG	BovineHD0100020157	1	69.85	0.0962	SNX4	37.98(U)	2.04E−07	−2.4836
FTLEG	ARS–BFGL–NGS−56584	1	145.09	0.1309	POFUT2	Intron	7.56E−08	2.0359
FTLEG	BovineHD0300019080	3	63.66	0.1248	ADGRL2	511.07(D)	1.06E−08	2.5317
FTLEG	BTB−01326707	6	38.00	0.2737	LCORL	665.01(D)	3.16E−11	−2.0018
FTLEG	BTB−00124923	9	34.94	0.1851	FRK	243.36(D)	3.42E−07	1.7253
FTLEG	BovineHD1300012605	13	42.81	0.4622	PYGB	0.61(D)	2.23E−09	1.6065
FTLEG	Hapmap50322–BTA−34017	13	78.20	0.1309	CEBPB	7.25(U)	8.11E−08	−2.2401
FTLEG	BovineHD1600000840	16	3.12	0.1191	KLHDC8A	11.96(D)	3.74E−07	2.1807
FTLEG	BovineHD1600008381	16	28.91	0.1683	TMEM63A	1.14(D)	7.79E−09	−2.1564
FTLEG	BovineHD2000011811	20	41.04	0.3221	SUB1	26.79(U)	4.00E−11	−1.9453
FTLEG	BTA−14388–rs29023151	22	23.20	0.4561	IL5RA	Intron	8.59E−10	1.6383

^*^U, Upstream; D, Downstream.

This study detected 10, 7, and 11 significant SNPs related to BFCI, DCCI, and FTLEG, respectively. There were 4 SNPs distributed on BTA 16. Three SNPs were found to be possibly as pleiotropism SNPs, including BovineHD4100004660 (BTA 6:38.22 Mb), BovineHD1300012605 (BTA 13:42.81 Mb) and BTA-50244-no-rs (BTA 20:34.30 Mb), respectively. Of these significant SNPs, the BTA-50244-no-rs SNP related to BFCI (*P* = 5.84E-13) was located downstream of the *PTGER4* gene.

### QTL annotation analysis

The LD of cows decreases with the increase of distance, when the distance is extended to 200 Kb, the decline rate of LD of cows tends to be gentle, and the average r^2^ value of cows is 0.3 at this time (show as Figure 2). The 100 Kb range of SNP upstream and downstream of significant trait association obtained from genome-wide association analysis is compared with the data that has been verified in the current cattle QTL database. Our significant SNPs associated with MY, FY, FP, PP and SCS overlapped with 1332, 1177, 3042, 1288, 24 QTLs, respectively. But there are also very few QTLs about body conformation traits overlapped with significant SNPs.

## Discussion

### Comparison with the other GWAS studies

In this study, FarmCPU was applied for screening the QTLs related to the milk production traits, health traits, and body conformation traits. A total of 95 significant SNPs were detected, located on the 93 candidate genes. Of these genes, *EHHADH, SLC37A1, PLEKHA5, TONSL, PLEC*, and *IL5RA* were reportedly related to milk production traits in other studies (15, 20, 21, 24, 39). However, this study did not detect some important candidate genes, such as *DGAT1*. Because in this study, the closest SNP on both flanks of *DGAT1* are BovineHD1400000206 (109.2 kb) and ARS-BFGL-NGS-55227 (50.8 Mb), respectively. Of these, BovineHD1400000206 associated with fat yield (*P* value = 2.76E−17). But the nearest gene on this significantly SNP is the TONSL gene (1.65 kb), which is a neighboring gene to *DGAT1*. So, the *DGAT1* gene was not detected in this study. The study by Ning et al. (40) used two models and a 70k SNP chip based on the Chinese Holsteins population and identified the *DGAT1* gene to be related to milk (40). Kim et al. (41) also obtained *DGAT1* affecting MY and FY in the Korean cattle population (41). Cole et al. (42) identified the *PHKA2* gene to be highly significant for four body size traits (stature, strength, body depth, rump width) (42). The 770k BeadChip was used by An et al. (43) to identify five candidate genes (*CSMD3, LAP3, SYN3, FAM19A5*, and *TIMP3*) related to the body conformation traits. This study did not detect the above genes to be associated with body conformation traits.

These inconsistencies might be due to differences in the detection platforms or algorithms used in the corresponding analysis, changes in the genetic background of the analyzed cattle, differences in the size and structure of the study population, or random or technical errors in some analyses. This also indicated that there are many important genetic markers or candidate genes in the bovine genome that are yet to be discovered.

### Genetic analysis of pleiotropic genes

Organisms have hundreds of thousands of genes and tens of thousands of phenotypes. The relationship between genes and epigenetic factors is complex. There are various associations such as pleiotropism, multigenic effect, polygene effect and so on. Pleiotropy is defined as the phenomenon where a single locus affects two or more distinct phenotypic traits (44, 45). It is common in nature. For example, the *DGAT1* gene is related to milk yield (40) and fat yield (26, 41). The genes *PIK3R6* and *PIK3R1* showed direct functional associations with height and body size (10). Production and health constitute fundamental dairy functions while body conformation traits are related to the functionality of the cow's body. So, the milk production traits and body conformation traits of dairy cows tend to complement each other. Certain identified regions related to conformation traits overlap with the performance traits such as reproduction (46), and milk production (47). Some genes in these regions were also involved in regulating the cell cycle or cell division, homeostasis, and lipid metabolism (10).

This study also reported this interesting phenomenon where the *PLEC, PLEKHA5*, and *TONSL* genes were found to belong to the pleiotropism gene for milk traits, and the *LCORL*, and *PTGER4* were pleiotropic genes for the body conformation traits. The *PLEC* gene (Plectin) can interlink different elements of the cytoskeleton. The *PLEC* gene was found to be associated with multiple traits, like MY, FP, and PP. Dan Wang et al. (20) also detected *PLEC* to have potential effects on the MY, FP, and PP traits, which could be useful for molecular breeding for milk production in Chinese Holsteins. The *PLEKHA5* gene, located on BTA 5, was predicted to enable the activity of binding phosphatidylinositol phosphate (48). Jiang et al. (21) showed the *PLEKHA5* gene to be significantly associated with FP using two different methods using 294,079 Holstein cows. The TONSL protein was considered to be an NF-κ negative regulator of B mediated transcription. Peters et al. (24), Nayeri et al. (25), and Atashi et al. (49) found this gene to be related to milk yield and the *TONSL* gene was found to reportedly affect the gamma-linolenic acid, long-chain saturated fatty acids and milk fat percent of the Canadian Holstein cows (26). Interesting, the *TONSL* gene is a neighboring gene to *DGAT1* (flanking <200 kb), associated with the fat percentage of milk (26).

Some studies on the *LCORL* gene showed it to affect human height (27), pig body length (28), horse height (29), chicken carcass weight (30), and the growth and development of cattle (31). This gene might have been a novel loci associated with the human skeletal frame size (32). *PTGER4* encodes a protein that is one of the members of the G-protein coupled receptor family, which imparts relaxation to the smooth muscle (34), leading to the phosphorylation of glycogen synthase kinase-3 (35), involved in osteoporosis (36), and regulating lipid droplet size and mitochondrial activity in the white adipose tissue (37, 38).

### Important candidate genes related to the fat metabolism or mammary gland development

Fatty acids are essential components of milk with known positive associations with human cardiovascular diseases and so on. This study identified genes such as *GPAT3, ARNTL2, EHHADH, CEBPB, DNAJB9, ZNF496, AGO2, GALNT18*, and *NEGR1 as* critical for obesity traits or adipose metabolism (see Table 7).

**Table 7:** Important candidate genes related to the fat metabolism or mammary gland development.

**Gene name**	**Location (BTA:Start–End, Mb)**	**Full name**	**Gene function**
GPAT3	6:98.29–98.36	Glycerol−3–phosphate acyltransferase 3	Highly expressed in the adipose tissue with an important role in adipogenesis (50). Can be regulated by folic acid for controlling lactation and metabolic function of the dairy cows (51). Involved in fat and lipid metabolism in the Yunling cattle (52).
ARNTL2	5:82.47–82.55	Aryl hydrocarbon receptor nuclear translocator like 2	Influencing Mexican–Mestizo childhood obesity (53).
EHHADH	1:81.88–81.93	Enoyl–CoA hyd ratase and 3–hydroxyacyl CoA dehydrogenase	Involved in fatty acid oxidation is essential for producing medium–chain dicarboxylic acids (54). Impact on the characteristics of milk fatty acid traits in Chinese Holstein (55). A pivotal gene in the fat–related pathway (56).
CEBPB	13:78.20–78.21	CCAAT enhancer binding protein beta	Involved in regulating the expression of fatty acid synthase in dairy cow mammary epithelial cells and milk fat synthesis (57).
DNAJB9	4:59.58–59.59	DnaJ heat shock protein family (Hsp40) member B9	The prognostic biomarkers of breast cancer (58). Correlated with the abdominal fat weight (59).
ZNF496	7:40.57–40.61	Zinc finger protein 496	Associated with milk fat and fertility (60).
AGO2	14:3.06–3.14	Argonaute RISC catalytic component 2	Related to mitochondrial oxidation and obesity–associated pathophysiology (61).
GALNT18	15:41.06–41.42	Polypeptide N–acetylgalactosaminyltransferase 18	Associated with milk protein and fat traits (62).
NEGR1	3:72.81–73.84	Neuronal growth regulator 1	Associated with obesity and BMI (body mass index) (63–65).
SLC37A1	1:142.81–142.87	Solute carrier family 37 member 1	Over–expressed in the bovine mammary tissue (66). Increases milk yield, decreases phosphorus concentration (66).
FNDC3B	1:95.12–95.41	Fibronectin type III domain containing 3B	Biomarker for the bovine mammary stem/progenitor cells, and Essential for the growth and maintenance of the mammary epithelium (67).

U, Upstream; D, Downstream.

*GPAT3* is highly expressed in the adipose tissue with an important role in adipogenesis (50). This gene can be regulated by folic acid for controlling lactation and metabolic function of the dairy cows (51) and is also involved in fat and lipid metabolism in the Yunling cattle (52). *EHHADH* involved in fatty acid oxidation is essential for producing medium-chain dicarboxylic acids (54). Hence, this gene has a key impact on the characteristics of milk fatty acid traits in Chinese Holstein (55). In porcine adipogenesis, *EHHADH* has been proposed to be a pivotal gene in the fat-related pathway (56). The *DNAJB9* gene is reportedly one of the prognostic biomarkers of breast cancer (58). Interestingly, DNAJB9 and DNAJB6 are members of the DNAJ gene family, with sequence similarity. The expression level of DNAJB6 in the chicken abdominal adipose tissue was significantly negatively correlated with the abdominal fat weight (59). *ZNF496* is reportedly associated with milk concentration (milk fat) and fertility (60). According to Gao et al. (62), the *GALNT18* gene was associated with milk protein and fat traits.

According to the known gene functions, some candidate genes were expressed in the mammary gland, such as the *SLC37A1*, and *FNDC3B* genes (see Table 7). *SLC37A1*, over-expressed in the bovine mammary tissue relative to the 17 other tissue types (66) transports glucose-6-phosphate in one direction and phosphorus in the other (68). Glucose is known to be essential for lactose synthesis in mammary cells. Kemper et al. (66) identified the causative mutation increasing the expression of SLC37A1 leading to an increase in milk yield and decreasing the phosphorus concentration.

### QTLs result overlapped with GWAS

Although many quantitative trait loci (QTLs) related to economically important traits in dairy cows have been identified, due to insufficient sample size and insufficient marker density used in QTL mapping research in history, not all genetic variations of these traits have been captured (69), in the study, we used GWAS to analyze the milk production traits, body conformation traits and somatic cells of dairy cows, and most of the results were also verified in the QTL analysis of dairy cows. Interestingly, our study found many SNP related to pleiotropy, but no repeated QTL regions were found in the QTL analysis (70). Also found the same phenomenon in the study of multiple traits of beef cattle. With these results, we can get some inspiration in verifying QTLs of some characteristics of interest shared among varieties (71).

## Conclusions

A total of 95 significant SNPs were identified to be related to the milk production, somatic cell score, and body conformation traits in Holstein cattle. Among them, 7 significant SNPs located on the *PLEC, PLEKHA5, TONSL, PTGER4*, and *LCORL* genes showed pleiotropic effects on milk production or body conformation traits. In addition, some important candidate genes, including *GPAT3, CEBPB, AGO2, SLC37A1*, and *FNDC3B*, were also found to be related to the fat metabolism or involved in mammary gland development. The above genes however need to be consolidated as new potential genes through future validation.

## Data availability statement

The original contributions presented in the study are included in the article or supplementary material, the variation data reported in this article have been deposited in the Genome Variation Map (GVM) in Big Data Center, Beijing Institute of Genomics (BIG), and Chinese Academy of Sciences, under accession numbers GVM000388 that are publicly accessible at https://bigd.big.ac.cn/gvm/getProjectDetail?project=GVM000388. The Bioproject accession number is PRJCA011726. Further inquiries can be directed to the corresponding author.

## Ethics statement

The animal study was reviewed and approved by Animal Care Advisory Committee, Northeast Agricultural University (Harbin, China).

## Author contributions

ZW, PW, and XL conceived the study and participated in its design. YZ, JW, QK, and XN were involved in the acquisition of data. XL, JW and QZ performed all data analysis. XL and ZW drafted the manuscript. ZW, PW, XL, YZ, CZ, QK, and XN contributed to the writing and editing. All authors read and approved the final manuscript.

## Funding

This work was financially supported by the Natural Science Foundation of China (No. 32070571), the Academic Backbone Project of Northeast Agricultural University (No. 15XG14), and the NEAU Research Founding for Excellent Young Teachers (2010RCB29).

## Conflict of interest

The authors declare that the research was conducted in the absence of any commercial or financial relationships that could be construed as a potential conflict of interest.

## Publisher's note

All claims expressed in this article are solely those of the authors and do not necessarily represent those of their affiliated organizations, or those of the publisher, the editors and the reviewers. Any product that may be evaluated in this article, or claim that may be made by its manufacturer, is not guaranteed or endorsed by the publisher.
